# An Improved DNA Extraction Method for Efficient and Quantitative Recovery of Phytoplankton Diversity in Natural Assemblages

**DOI:** 10.1371/journal.pone.0133060

**Published:** 2015-07-28

**Authors:** Jian Yuan, Meizhen Li, Senjie Lin

**Affiliations:** 1 The State Key Laboratory of Marine Environmental Science, Xiamen University, Xiamen, Fujian, China; 2 Marine Biodiversity and Global Change Research Center, Xiamen University, Xiamen, Fujian, China; 3 The Department of Marine Sciences, University of Connecticut, Groton, Connecticut, United States of America; Deutsches Krebsforschungszentrum, GERMANY

## Abstract

Marine phytoplankton are highly diverse with different species possessing different cell coverings, posing challenges for thoroughly breaking the cells in DNA extraction yet preserving DNA integrity. While quantitative molecular techniques have been increasingly used in phytoplankton research, an effective and simple method broadly applicable to different lineages and natural assemblages is still lacking. In this study, we developed a bead-beating protocol based on our previous experience and tested it against 9 species of phytoplankton representing different lineages and different cell covering rigidities. We found the bead-beating method enhanced the final yield of DNA (highest as 2 folds) in comparison with the non-bead-beating method, while also preserving the DNA integrity. When our method was applied to a field sample collected at a subtropical bay located in Xiamen, China, the resultant ITS clone library revealed a highly diverse assemblage of phytoplankton and other micro-eukaryotes, including Archaea, Amoebozoa, Chlorophyta, Ciliphora, Bacillariophyta, Dinophyta, Fungi, Metazoa, etc. The appearance of thecate dinoflagellates, thin-walled phytoplankton and “naked” unicellular organisms indicates that our method could obtain the intact DNA of organisms with different cell coverings. All the results demonstrate that our method is useful for DNA extraction of phytoplankton and environmental surveys of their diversity and abundance.

## Introduction

Marine phytoplankton are ubiquitous in the global marine environment and constitute the largest part of primary producers in the ocean. Many coastal phytoplankton species form harmful algal blooms and some of them produce toxins (e.g. PSP, DSP), causing devastating impacts to the coastal marine environment and public health concerns [1]. Accurately identifying the species and quantifying the abundance of each species over space and time, which are essential for understanding ecological behaviors of the assemblages, often require molecular techniques. For quantitative molecular analyses, efficiently extracting high-quality DNA from phytoplankton is essential. Although many studies have employed molecular techniques, most of them adopt protocols from model organisms with various modifications. The protocol optimization for each species is very time consuming and using different protocols often complicates result comparison. It is highly desirable to develop a standardized protocol.

One of the major sources of variability in DNA extraction efficiency comes from different cell coverings in different phytoplankton species that may present different degrees of difficulties in lysing the cells completely. For example, armored dinoflagellates have tough thecae and some athecate dinoflagellates (e.g. *Symbiodinium* spp.) and *Chlorella* spp. can be very resilient to harsh lytic incubation. Some physical processing is needed to help disrupt the cells of these species. Repeated freezing-thawing can enhance cell breakage and has been widely used due to its simplicity [2]. Although the cell wall is tough, it could become delicate under extreme low temperatures such as in liquid nitrogen. Thus, grinding cells in liquid nitrogen is efficient in breaking the hard covering of species such as armored dinoflagellates [3]. Because the ultrasonic waves can easily destroy the cell coverings and DNA as well, sonication is used in DNA extraction less than other cellular components such as proteins and pigments [4].

Recently, bead-beating has become increasingly used for disrupting phytoplankton cells [5, 6, 7, 8, 9, 10]. The high-speed agitation of samples mixed with beads made of different materials (e.g. ceramic, glass, zirconia, etc.) can disrupt any kind of cells in water or sediment samples. It has been reported that bead-beating leads to higher DNA yield compared with other methods [11]. However, the method has not been systematically examined for different species. Moreover, whether DNA extracted from the bead-beating method is intact enough to allow quantitative measurements of gene copies remains to be investigated.

In this study, we developed a simple and effective protocol of DNA extraction from phytoplankton. Nine species of phytoplankton were examined for the efficiency in DNA extraction, including species that are known for the difficulty in cell disruption (*Alexandrium fundyense*, *Prorocentrum donghaiense* and *Chlorella* sp.). We also examined whether the DNA obtained was intact enough for quantitative measurement of ITS (Internal Transcribed Spacer) for laboratory cultures mixed with field sample. Finally, we demonstrated that this method was applicable to environment samples by analyzing a subtropical plankton assemblage from Wuyuan Bay, Xiamen, China.

## Materials and Methods

### Algal cultures and sample collection

We selected 9 species from five phyla to investigate the effectiveness of our method for a wide range of phytoplankton (Table 1). Although only 9 species were used in this study, they represented commonly occurring species and species with different toughness for cell breakage. These species were cultured in f/2 or f/2-Si medium prepared with 0.2-μm filtered and autoclaved seawater, with salinity adjusted to 30 ppt, at 20°C and a 14: 10 h light-dark cycle with a photon flux density of 80 μmol·m^-2^·s^-1^. When the cultures reached the exponential growth phase, two sets of samples containing 15 replicates each were collected for the 9 species respectively: one set for DNA extraction by bead-beating and the other without bead-beating. The 50-mL samples were centrifuged at 4500 x g in an Eppendorf centrifuge 5804R (Eppendorf, Germany) at 15°C for 15 min. The cell pellet was then suspended in about 1 mL medium and pipetted into a 2 mL tube and centrifuged again at ~9391 x *g* in an Eppendorf centrifuge 5424 (Eppendorf, Germany) for 10 min. After supernatant was removed, the cell pellet was resuspended thoroughly in 1 mL DNA lysis buffer (10mM Tris-HCl, pH 8.0; 100 mM EDTA, pH 8.0; 0.5% w/v SDS) and proceeded to DNA extraction.

**Table 1:** The nine phytoplankton species used in this study.

Species	Phylum	Medium	Source	#Cells used
*Alexandrium fundyense*	Dinophyta	f/2-Si^a^	CCMP^b^ 1719	6.3 × 10^4^
*Karenia mikimotoi*	Dinophyta	f/2-Si	Daya Bay, Guangdong	5.1 × 10^5^
*Prorocentrum donghaiense*	Dinophyta	f/2-Si	Shengsi Islands, Zhejiang	3.2 × 10^6^
*Skeletonema costatum*	Bacillariophyta	f/2	Xiamen Harbor, Fujian	6.8 × 10^6^
*Chaetoceros muelleri*	Bacillariophyta	f/2	Xiamen Harbor, Fujian	5 × 10^7^
*Thalassiosira weissflogii*	Bacillariophyta	f/2	Daya Bay, Guangdong	6.3 × 10^6^
*Isochrysis galbana*	Haptophyta	f/2-Si	Xiamen Harbor, Fujian	2.6 × 10^7^
*Chlorella* sp.	Chlorophyta	f/2-Si	Dongshan Island, Fujian	6.8 × 10^7^
*Heterosigma akashiwo*	Raphidophyta	f/2-Si	Changjiang Estuary	1.7 × 10^6^

^a^ f/2-Si medium is f/2 medium without Na_2_SiO_3_.

^b^ CCMP refers to Provasoli-Guillard National Center for Marine Algae and Microbiota.

### DNA isolation and purification

Ten μL Proteinase K (200 μg/mL) was pipetted into each sample, and after brief vortexing, the sample was incubated at 55°C for 3 days. The 3-day incubation was determined depending on the results of preliminary experiments in which it was demonstrated that 3 days was the most appropriate time span (S2 Fig). When the incubation was completed, 2 μL of the lysate was removed and observed under a Nikon YS100 microscope (Nikon, Japan) to assess degree of cell breakage. The non-bead-beating samples were directly subjected to DNA extraction (next paragraph). The bead-beating sets of samples were then centrifuged at ~13,523 x *g* on an Eppendorf centrifuge 5424 (Eppendorf, Germany) for 10 min, and 800 μL of the supernatant was removed into a new tube. Next, approximately 0.05–0.15 g (depending on the pellet size) of 0.5 mm diameter ceramic beads were added into each tube. The samples with beads were loaded onto a FastPrep-24 bead mill (MP Biomedicals, USA) for bead-beating at 6 m/s for 1 min, performed three times with 1 min intervals when the samples were placed on ice. When bead-beating was completed, 2 μL of the lysate was observed under the microscope, and each sample was mixed with its corresponding 800 μL supernatant removed earlier and incubated at 55°C for 30 min.

The subsequent DNA extraction was carried out following our reported protocol [12, 13]. Briefly, 165 μL of 5M NaCl solution was pipetted into each sample, followed by addition of 165 μL of 10% w/v CTAB (Cetyl/Hexadecyl Trimethyl Ammonium Bromide, in 0.7 M NaCl solution) and incubation at 56°C for 10 min. Each sample was split equally into two. To each, 665 μL of chloroform were added and samples were vortexed. The samples were centrifuged at ~13,523 x *g* for 10 min for separation of liquid layers, and the upper layer was carefully transferred into new tubes. The following procedure of purification was implemented with DNA Clean & Concentrator kit (Zymo Research, USA): binding buffer (2x volume of the former upper layer) was pipetted into each tube, and the mixture was loaded into the columns and centrifuged at ~13,523 x *g* for 30 s; next, the columns were loaded with 200 μL of wash buffer and centrifuged by ~13,523 x *g* for 30 s, and repeated once; finally, DNA was eluted in 100 μL of 10 mM Tris-HCl by centrifugation of ~13,523 x *g* for 1 min. The concentrations and purities of DNA were measured on a NanoDrop 2000 Spectrophotometer (Thermo Scientific, USA). DNA contents (pg/cell) for each replicate were determined using their DNA masses divided by corresponding cell numbers. DNA integrity was generally assessed by gel electrophoresis and the images were captured on a Bio-Rad ChemiDoc XRS+ gel imager (Bio-Rad Inc., USA).

### Examination of DNA integrity by cloning ITS region and qPCR

To accurately assess the integrity of DNA, we used qPCR to compare the copies of ITS from the same amount of DNA extracted using the bead-beating and non-bead-beating methods. A pair of previously reported eukaryotic universal primers (18ScomF-3end: GTCGTAACAAGGTTTCCGTAGGTG; com28SR2: TTAGACTCCTTGGTCCGTGTTT) was used to amplify an rDNA region from the 3’-end of 18S to 5’-end of 28S containing the whole ITS region (ITS1-5.8S-ITS2) [14]. DNA obtained by bead-beating method was used as templates. The *Ex Taq* PCR kit (Takara Bio, Japan) was used following instructions with final concentration of primers at 0.2 μM. The PCR program was as follows: pre-denaturation at 94°C for 5 min; 35 cycles of 95°C for 30 s, 56°C for 30 s, 72°C for 1 min 30 s,; 72°C for 5 min. Then the PCR products were ligated to pMD 19-T vectors (Takara Bio, Japan) and cloned. Plasmids were isolated from positive (white) clones and the insert was PCR amplified with vector primers M13F and M13R (PCR procedures as above, except annealing temperature at 55°C). All PCR assays were implemented on a Bio-Rad T100 Thermal Cycler (Bio-Rad Inc., USA) and these PCR products were purified and used as standard samples for quantification of ITS copies in the following qPCR experiments, because there would be serious overestimation using circular plasmids as standard in qPCR [15].

Specific primers (Table 2) were designed using Primer Premier 5 based on the ITS sequences of the 9 species we obtained. They were used in qPCR on a Bio-Rad CFX96 Real-Time System and C1000 Thermal Cycler (Bio-Rad Inc., USA). The FastStart Universal SYBR Green Master (ROX) (Roche Applied Science, Germany) was used as instructed and the final concentration of primers was 0.4 μM. The thermocycle consisted of pre-denaturation at 95°C for 10 min; 95°C for 20 s, T_a_ (Table 2) for 30 s, for a total 40 cycles; melt curve generation from 65°C to 95°C with increment of 0.5°C. The qPCR standard samples were serially diluted by 10 fold to yield 1−10^5^ dynamic range, and the DNA stock solution of the 9 algal species by both methods was also diluted into 10-fold series. The ITS copies per cell for each species were separately determined from each dilution and then averaged and compared.

**Table 2:** The sequences and annealing temperatures (T_a_’s) of the specific primers of ITS used in qPCR.

Species	Primers	Sequence (5’- 3’)	T_a_ (°C)
*A*. *fundyense*	AfF	CATGGTTGATTGGGGGCA	52
	AfR	AAGTCTTCAGCTTGTCTCAGTTG	
*K*. *mikimotoi*	KmF	GCTCTTCCTGCCCCTGATG	54
	KmR	ATGGAAGGAAGCCACAAATG	
*P*. *donghaiense*	PdF	CCGTCTTCTGGGCTTGTCC	54
	PdR	AAAGTTGTAAGAAGATAAAGAGTGGAAG	
*S*. *costatum*	ScF	TATCCAAACCTTACTTCCCCGTG	53
	ScR	CGCCTGGTTTTGGTTTTTGTAAGT	
*C*. *muelleri*	CmF	GAGAGTAAGTTAGCAGCCG	53
	CmR	CTCGCAAAATGCCATCCAG	
*T*. *weissflogii*	TwF	GTGGAAGCCGCCTGAGACC	56
	TwR	GTATGCCGGAGAAGCTAGAGAC	
*I*. *galbana*	IgF	GTCTTTCCACCCCACACTG	55
	IgR	ACAATCGCAAACCCCAGAC	
*Chlorella* sp.	ChF	CCGCCTGGTAATTTTGTCC	53
	ChR	TGTCTTTTGTTTGTGGACGG	
*H*.*akashiwo*	HaF	CCGACGGGCGTGGTAGC	55
	HaR	TCCTCTGTCAGAACAACCGAAGT	

### Measurement of genome sizes of dinoflagellates by flow cytometry

To assess the extent of cell and DNA loss in the cell harvesting and DNA extraction process, we attempted to compare cellular DNA content of the 9 species estimated from our extraction protocol with that measured by flow cytometry. Because *S*. *costatum* is chain-forming and cells cannot be separated, flow cytometric measurement was not possible. The cells of *H*. *akashiwo* tended to aggregate after fixation in cold 100% methanol and could not be dispersed in PBS, its cellular DNA content was also not measured flow cytometrically.

Cells were collected by centrifugation at ~2880 x g for 10 min at 4°C, and the chlorophyll was extracted by resuspending the pellets in 1 mL of cold 100% methanol and stored overnight at 4°C. The cells were then washed twice in PBS (pH = 7.4) and the pellets were resuspended in 0.5 mL staining solution (50 μg propidium iodide mL-1 PBS) containing 0.2 mg/mL RNase A for at least 2 h before analysis. Chicken red blood cells (Genome size ~2.33 x 10^9^ base pairs or Gbp) were added as references for the 3 large-genome dinoflagellates. As the genome sizes of the other 4 species were too small to be measured properly with chicken red blood cells as the standard, a strain of *Thalassiosira pseudonana* (from the Changjiang Estuary, China) was used as an alternative standard, with its genome size being set to be the same as the estimate for strain CCMP 1335 (~0.035 Gbp) based on genome assembly [16].

Samples were run on Cell Lab Quanta SC flow cytometer (Beckman Coulter Inc., USA) equipped with 488 nm laser at a speed of ~18 μL/min. Data were acquired in linear and log mode until 20000 events had been recorded. The fluorescence emission of propidium iodide was detected at 575 nm. All the data analyses including peak numbers, coefficients of variations were carried out using the software FlowJo. DNA quantity corresponding to the G1 peak (the stage of the cell cycle with 1N amount of DNA) was recorded as the genome size.

### Testing of the method using field samples mixed with cultured samples

In order to examine whether this method is applicable in processing field samples, we mixed some cultured species into field samples and carried out qPCR. A plankton sample was collected in Wuyuan Bay (N24°31’45”, E118°10’53”; Xiamen, China) on September 14th 2013 with plankton net with 10 μm-pore size mesh (model 9000; Sea-Gear Corp., Melbourne, Florida, USA) and filtered through bolting cloth with mesh size of 100 μm to remove large particles. The field sampling required no specific permissions because this area is legally open to the public, and our research did not involve endangered or protected species. The resulting 10–100 μm sample was divided into several subsamples, which were separately filtered through polycarbonate membrane with pore size of 3 μm. Meanwhile, a subsample was fixed with Lugol’s solution for microscopic observation.

Since no *A*. *fundyense*, *P*. *donghaiense* and *Chlorella* sp. was observed in the field samples, we added 1.1 × 10^4^, 2.3 × 10^5^ and 2 × 10^7^ cells of these species from cultures respectively to one subsample and extracted DNA using our bead-beating method. The added cells were equivalent to 7.2 × 10^8^, 5 × 10^8^ and 5.2 × 10^8^ ITS copies by data obtained from culture samples earlier in our study. PCR was carried out with 18ScomF-3end and com28SR2 and the same procedures above. Sixty-seven white colonies were picked for sequencing after cloning of the PCR products, from which the ratio of sequences of the 3 species were estimated. The abundances of A. *fundyense*, *P*. *donghaiense* and *Chlorella* sp. in the mixed samples were also calculated by qPCR in order to verify whether they were equal to those actually used. The corresponding specific primers in Table 2 were used with the same qPCR procedures mentioned above.

### Application of the method to the analysis of a natural plankton assemblage

To assess the applicability of our protocol to biodiversity research for natural phytoplankton assemblages, surface seawater was collected from Wuyuan Bay on September 4th 2014. Following filtration through the bolting cloth with mesh size of 100 μm, the filtrate was filtered onto polycarbonate membranes with pore size of 3 μm and 0.2 μm successively. Then the DNA was extracted from the two samples (3–100 μm, 0.2–3 μm) and used separately as the template in PCR with primers 18ScomF-3end and com28SR2. The PCR procedure was the same as mentioned above. The products were also cloned, and 100 white colonies were picked and sequenced for each sample. The sequences were aligned using ClustalX2. Phylogenetic trees were inferred using both Neighbor Joining (NJ) and Maximum Likelihood (ML) methods with the evolutionary model “Tamura-Nei” selected based on ModelTest analysis result and with bootstrap replication of 1000. OTUs were preliminarily determined by the clades and relative distances shown in the tree. The sequences in each OTU were further aligned for the determination of final members of each OTU based on 98% sequence identity [17]. The most abundant sequence in each OTU was selected to represent the OTU for taxonomic annotation; these sequences were analyzed by BLAST and top hits were used to assign an appropriate taxonomic entity based on the Lowest Common Ancestor (LCA) method [18]. To obtain a more detailed profile of taxon composition, the top hit sequences were downloaded and aligned with our toxon-representing sequences by ClustalX2. The NJ and ML trees were carried out as described above with *E*. *coli* as the outgroup to root the tree.

### Statistical analysis

The bead-beating and non-bead-beating methods were compared using t-test. Because each sample for each species had 15 replicates and their variances were consequently different, independent two-sample t-test method concerning equal sample sizes and unequal variances was employed. This was implemented for comparisons on DNA contents, ITS copies per cell, and the ratios of DNA contents estimated using the two DNA extraction methods in each species, and the ratios of ITS copies quantified from DNA samples extracted using the two methods in each species. Probability (*p*) value of 0.05 was used as the criterion of statistical significance for the difference.

## Results

### Efficiency of DNA extraction and DNA integrity in cultured species

**Fig 1:**
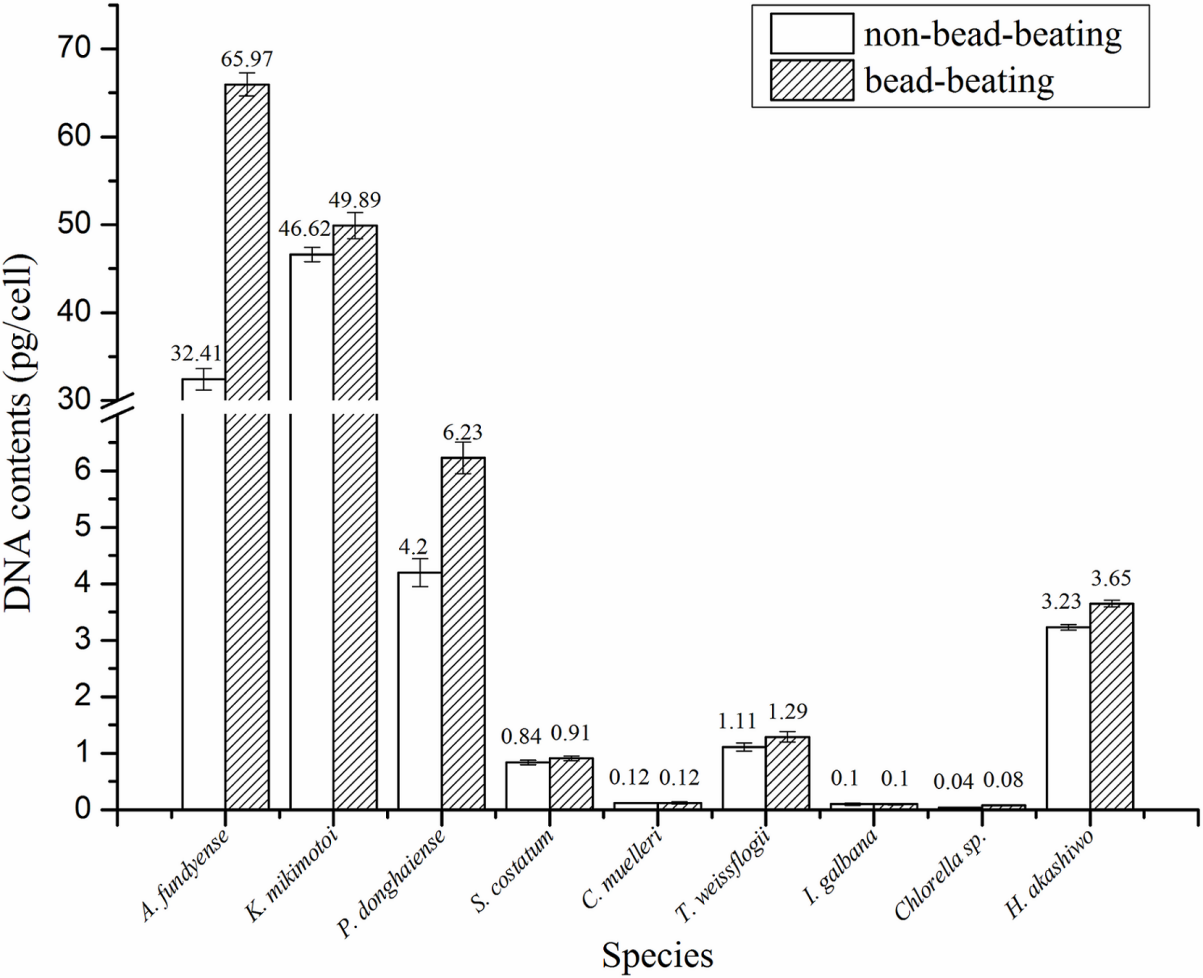
Comparison between bead-beating method and non-bead-beating method in estimated cellular DNA content. Shown are averages, with error bars indicating standard deviations (n = 15).

After incubation at 55°C for 3 days but without bead-beating, intact cells with discernable intracellular components were found under the microscope in the samples of *A*. *fundyense*, *P*. *donghaiense* and *Chlorella* sp., although no intact cells were noticeable for other species. No intact cells were observed in any species after bead-beating. As shown in Fig 1, the DNA yields increased for nearly all the species as a result of the use of bead-beating. Particularly, the yields of DNA increased markedly (~1.48 to 2.04-fold) for *A*. *fundyense*, *P*. *donghaiense* and *Chlorella* sp. There were significant increases (*p* < 0.05) in the DNA yields for all the 9 species except *C*. *muelleri* and *I*. *galbana* (Table 3).

**Table 3:** The comparisons of DNA yields and ITS copies between bead-beating and non-bead-beating methods for the 9 phytoplanktonic species (t-test).

Species	*p* values	
	DNA yields	ITS copies
*A*. *fundyense*	3 × 10^−33^	1.74 × 10^−33^
*K*. *mikimotoi*	4.16 × 10^−8^	1.29 × 10^−3^
*P*. *donghaiense*	1.53 × 10^−18^	5.63 × 10^−31^
*S*. *costatum*	1.68 × 10^−5^	1.54 × 10^−5^
*C*. *muelleri*	0.48	0.4
*T*. *weissflogii*	2.06 × 10^−6^	1.3 × 10^−5^
*I*. *galbana*	0.92	0.1
*Chlorella* sp.	1.09 × 10^−25^	8.05 × 10^−25^
*H*. *akashiwo*	4.63 × 10^−19^	4.41 × 10^−7^

**Fig 2:**
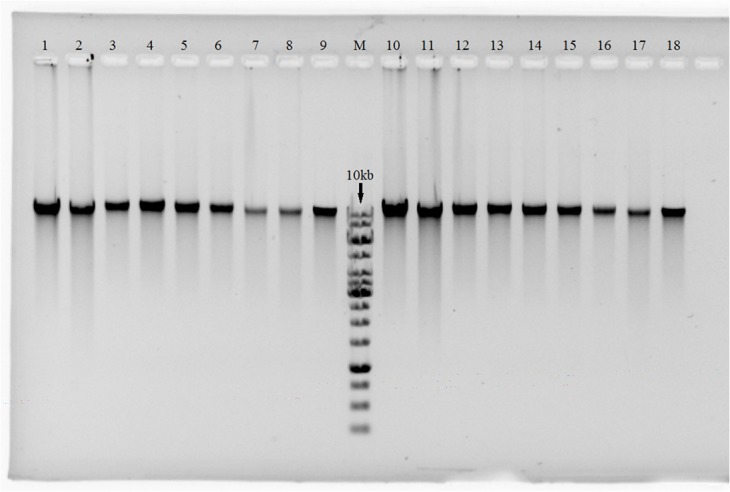
Gel electrophoretic result of extracted DNA indicating similar DNA integrity between bead-beating (right of lane M) and non-bead-beating (left of lane M) methods. Lanes 1, 10: *A*. *fundyense*; 2,11: *K*. *mikimotoi*; 3, 12: *P*. *donghaiense*; 4,13: *S*. *costatum*; 5,14: *C*. *muelleri*; 6,15: *T*. *weissflogii*; 7,16: *I*. *galbana*; 8,17: *Chlorella* sp.; 9,18: *H*. *akashiwo*. M is GeneRuler 1kb DNA Ladder (Thermo Scientific, USA) with the largest size of 10 kb.

Gel electrophoresis of the extracted DNA samples showed similar band patterns between bead-beating and non-bead-beating methods, indicating that the DNA extracted using the bead-beating method was not less intact than that by the non-bead-beating method (Fig 2). The fact that all visible bands were longer than 10 kb indicated sufficient integrity of DNA for PCR amplification and many other applications. Furthermore, the A260/A280 and A260/A230 ratios of the DNA by the bead-beating method were 1.8–2.0 and 2.0–2.2, respectively (Table 4), indicating good quality of the DNA extracts following our purification protocol.

**Table 4:** Comparison of DNA purities resulting from the two DNA extraction methods indicated by A260/A280 and A260/A230 ratios (mean ± standard deviation; n = 15).

Species	Non-bead-beating		Bead-beating	
	A260/A280	A260/A230	A260/A280	A260/A230
*A*. *fundyense*	1.85 ± 0.04	1.96 ± 0.02	1.94 ± 0.03	2.05 ± 0.04
*K*. *mikimotoi*	1.92 ± 0.01	2.2 ± 0.03	1.94 ± 0.01	2.12 ± 0.02
*P*. *donghaiense*	1.93 ± 0.02	2.14 ± 0.01	1.93 ± 0.02	2.09 ± 0.04
*S*. *costatum*	1.89 ± 0.02	2.17 ± 0.03	1.85 ±0.04	2.04 ± 0.04
*C*. *muelleri*	1.8 ± 0.02	2.04 ± 0.04	1.81 ± 0.02	2.08 ± 0.01
*T*. *weissflogii*	1.88 ± 0.03	2.12 ± 0.03	1.91 ± 0.03	2.04 ± 0.05
*I*. *galbana*	1.84 ± 0.02	2.03 ± 0.04	1.9 ± 0.03	2.02 ± 0.03
*Chlorella* sp.	1.92 ± 0.02	2.1 ± 0.05	1.89 ± 0.03	2.03 ± 0.02
*H*. *akashiwo*	1.87 ± 0.02	2.08 ± 0.03	1.88 ± 0.01	2.06 ± 0.02

### Isolation of ITS region and quantification of copy number for cultured species

**Fig 3:**
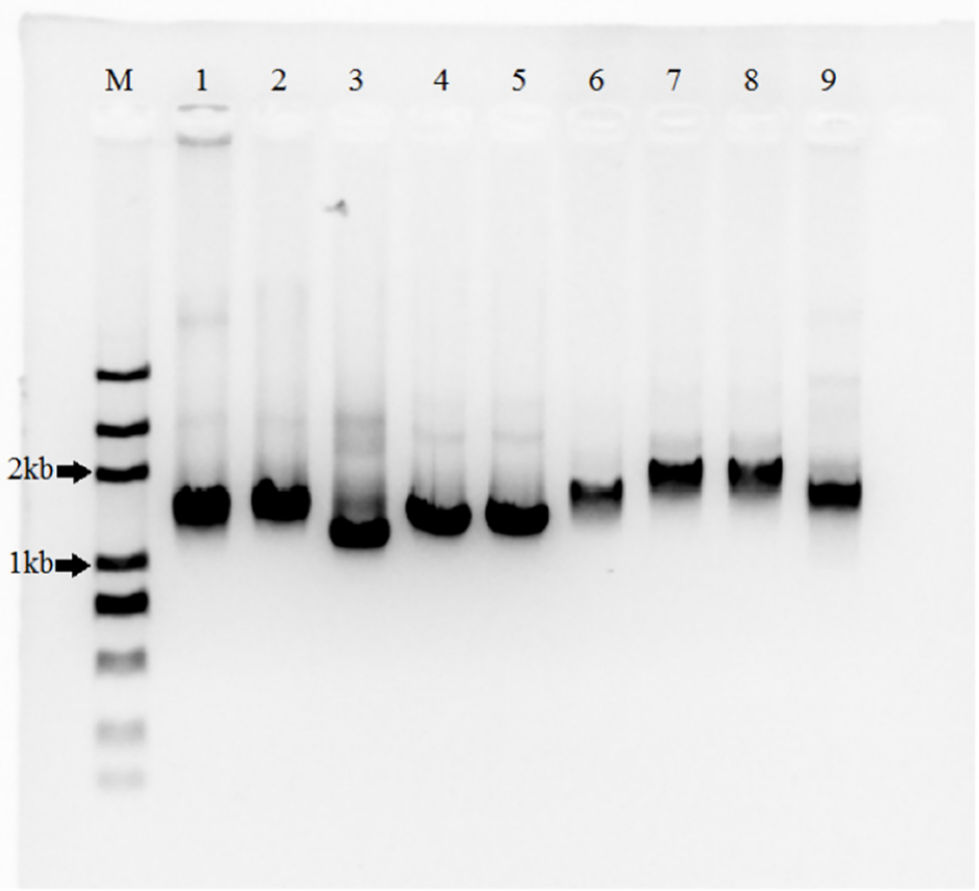
The amplification of the whole ITS region using primers 18ScomF-3end and com28SR2 with bead-beating extracted DNA as template. Different sizes of the amplicons reflected different lengths of the ITS region among different species. Lanes 1–9, *A*. *fundyense*, *K*. *mikimotoi*, *P*. *donghaiense*, *S*. *costatum*, *C*. *muelleri*, *T*. *weissflogii*, *I*. *galbana*, *Chlorella* sp., *H*. *akashiwo*, respectively. Lane M, DL2000 DNA Marker (Takara Bio, Japan).

**Fig 4:**
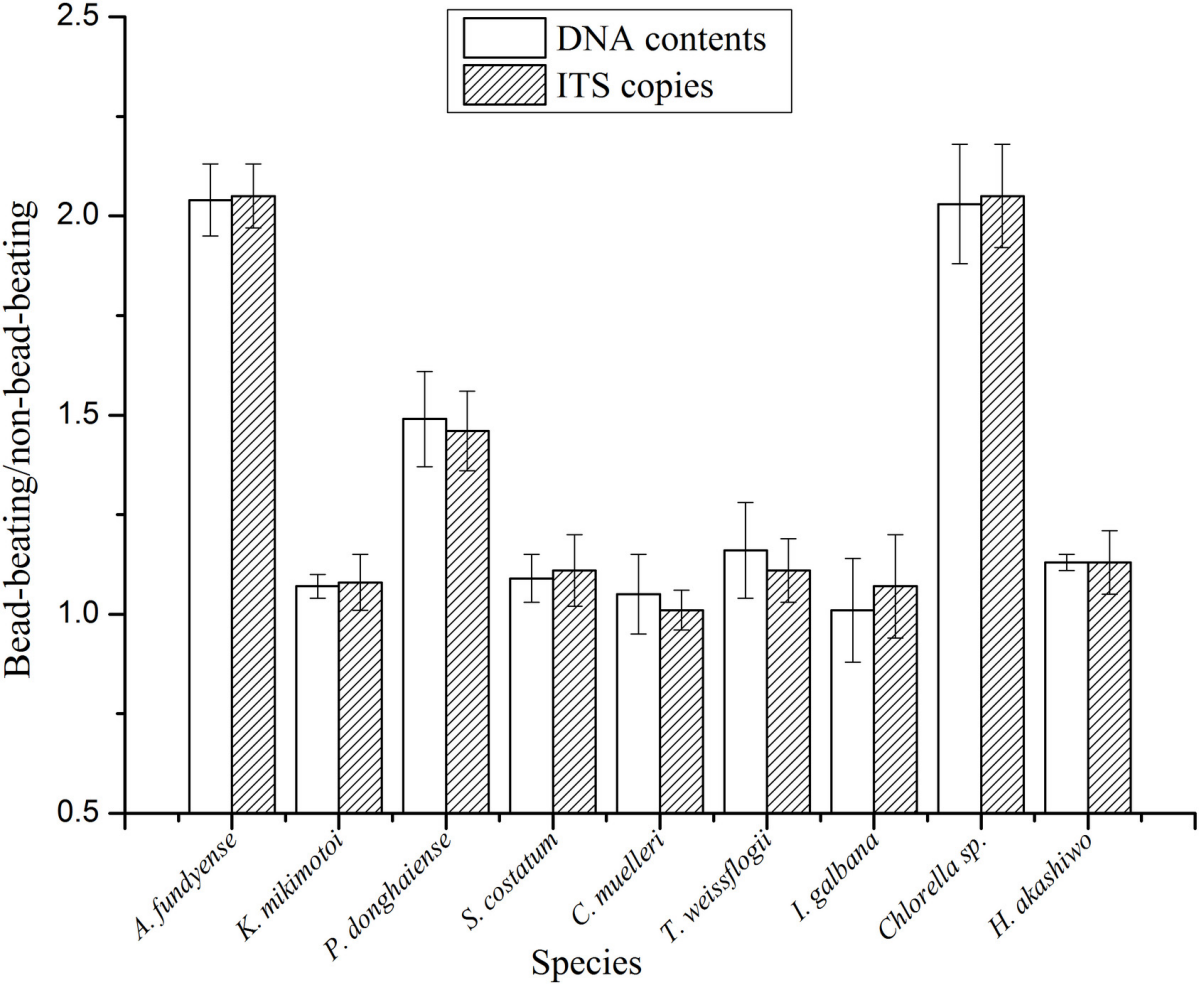
Comparison of bead-beating with non-bead-beating methods in terms of DNA contents and ITS copy number. Shown are averages, with error bars indicating standard deviations (n = 15). No significant difference was found between the ratios of DNA contents and those of ITS copies in any of the species examined (*p* > 0.05, t-test).

The amplification of the ~1.5kb ITS region (spanning 3’ end of 18S, ITS1, 5.8S, ITS2, and 5’ end of 28S) was successful for all 9 species (Fig 3). The amplicons were sequenced and the previously undocumented sequences have been deposited at NCBI under the accession numbers KF998560–KF998568. With the sequences, specific primers of ITS were designed and qPCR was conducted for each species. Based on the number of cells collected for each sample for DNA extraction, qPCR results were translated to ITS copies per cell (Table 5). The estimates of the ITS copies for the samples by bead-beating method were clearly higher than were those by non-bead-beating method, especially in *A*. *fundyense*, *P*. *donghaiense* and *Chlorella* sp. that showed 2.05, 1.46 and 2.05-fold increase, respectively. As in the DNA yields, the ITS copies increased significantly (*p* < 0.05) for all the 9 species except *C*. *muelleri* and *I*. *galbana* (Table 3). Furthermore, these fold increases were not significantly different from those observed in DNA contents mentioned above (*p* > 0.05, Fig 4). All the results demonstrated that the DNA yields increased significantly for nearly all species including the thick-walled dinoflagellates, and the DNA integrity was preserved.

**Table 5:** ITS copies per cell calculated from qPCR results obtained from DNA templates prepared using the two DNA extraction methods (mean ± standard deviation; n = 15).

Species	Standard equation^a^	R^2^	ITS copies per cell	
			Non-bead-beating	Bead-beating
*A*. *fundyense*	y = -3.464x + 39.001	0.999	(3.213 ± 0.133) × 10^4^	(6.574 ± 0.21) × 10^4^
*K*. *mikimotoi*	y = -3.474x + 38.992	0.999	(3.441 ± 0.199) × 10^3^	(3.701 ± 0.2) × 10^3^
*P*. *donghaiense*	y = -3.569x + 41.754	0.998	(1.51 ± 0.088) × 10^3^	(2.199 ± 0.057) × 10^3^
*S*. *costatum*	y = -3.428x + 38.471	1	(4.093 ± 0.151) × 10^2^	(4.53 ± 0.287) × 10^2^
*C*. *muelleri*	y = -3.435x + 39.397	0.999	(1.04 ± 0.023) × 10^2^	(1.051 ± 0.039) × 10^2^
*T*. *weissflogii*	y = -3.519x + 40.47	0.999	(2.505 ± 0.111) × 10^2^	(2.777 ± 0.165) × 10^2^
*I*. *galbana*	y = -3.364x + 38.531	0.998	2.99 ± 0.33	3.18 ± 0.268
*Chlorella* sp.	y = -3.642x + 40.667	0.999	(1.289 ± 0.051) × 10	(2.637 ± 0.138) × 10
*H*.*akashiwo*	y = -3.406x + 38.253	1	(3.262 ± 0.211) × 10^2^	(3.668 ± 0.116) × 10^2^

^a^ In these equations, y is the threshold cycle numbers (C_t_) and x is the decimal logarithm of ITS copies

### Comparison of genome size estimates from flow cytometric analysis and DNA extraction

As shown in Table 6, for the three dinoflagellates, the DNA contents estimated from the final DNA yield and the number of cells used in DNA extraction were 75.76–81.23% of that estimated from flow cytometric analysis. These indicated 18.77–24.24% total loss of cells (during harvesting) and DNA extraction (in the purification process). For the other 4 species, the DNA contents based on DNA extraction exceeded that from flow cytometric analysis.

**Table 6:** Genome sizes (Gbp^a^) of the 7 phytoplankton species measured using flow cytometric and DNA extraction methods (mean ± standard deviation).

Species	Reference	Flow cytometry (n = 3)	DNA extraction (n = 15)
*A*. *fundyense*	Chicken red blood cells	84 ± 8.56	65.97 ± 1.33
*K*. *mikimotoi*	Chicken red blood cells	66.5 ± 3.58	49.89 ± 1.49
*P*. *donghaiense*	Chicken red blood cells	8.36 ± 0.03	6.23 ± 0.28
*C*. *muelluri*	*Thalassiosira pseudonana*	0.046	0.12 ± 0.02
*T*. *weissflogii*	*Thalassiosira pseudonana*	0.084 ± 0.012	1.29 ± 0.09
*I*. *galbana*	*Thalassiosira pseudonana*	0.041	0.1 ± 0.01
*Chlorella* sp.	*Thalassiosira pseudonana*	0.027 ± 0.003	0.08 ± 0.003

^a^ 1 Gbp ≈ 0.978 picogram or pg of DNA.

### Retrieval of absolute and relative abundances of cultured species mixed into field sample

For the mixed field samples, 100 clone sequences of ITS amplicon were obtained, which included 40 of *A*. *fundyense*, 26 of *P*. *donghaiense*, 26 of *Chlorella* sp., and 8 of environmental species. The ratio of the three added species (1.54: 1: 1) was similar to that predicted (1.44: 1: 1.04) based on the cell number of each species added to the field sample and ITS copy number estimated earlier using qPCR. Furthermore, the cell numbers estimated by qPCR were also close to those actually added (Table 7). Therefore, it was clear that our method was applicable in quantitative research of phytoplankton in field investigation.

**Table 7:** The number of cells and their ratios of the three algal species added into the field sample and those estimated using qPCR.

	*A*. *fundyense*	*P*. *donghaiense*	*Chlorella* sp.	Ratio
Added	1.1 × 10^4^	2.3 × 10^5^	2 × 10^7^	1.1: 23: 2000
Estimated	1.2 × 10^4^	2.4 × 10^5^	1.9 × 10^7^	1.2: 24: 1900

### Biodiversity of a natural plankton assemblage revealed using the protocol developed

**Fig 5:**
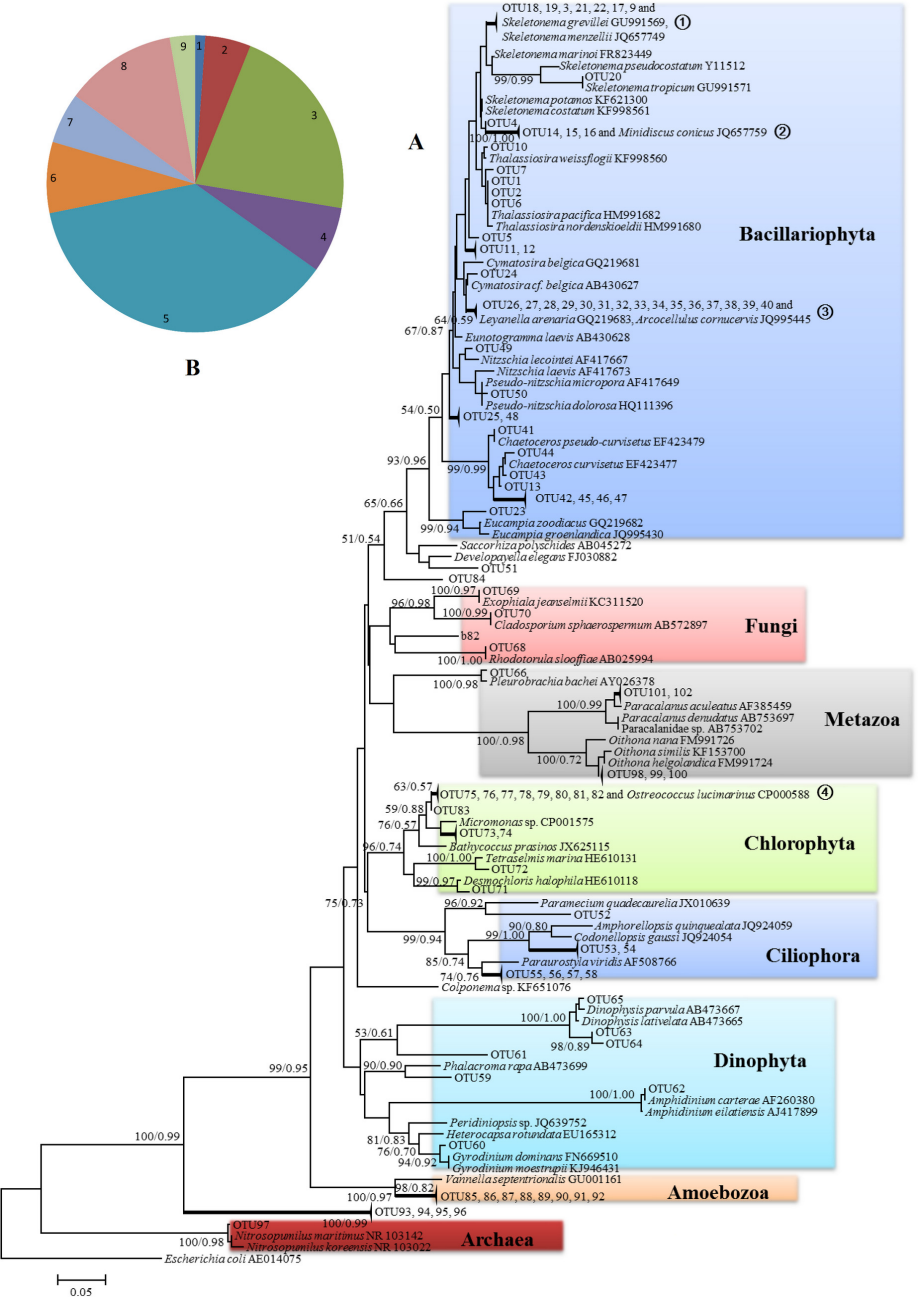
The phylogenetic affiliations (A) and taxa composition (B) of the field samples obtained from the retrieved rDNA clone library. In (A), values at the nodes are bootstrap values derived from Neighbor-joining and Maximum Likelihood methods (NJ/ML); only those larger than 50/0.50 are shown. The branches consisting of only our retrieved clones (OTUs) (except ①, ②, ③ and ④) were collapsed. In (B), 1–9 refer to Archaea (1.1%), Amoebozoa (4.97%), Chlorophyta (21.55%), Ciliophora (7.18%), Bacillariophyta (37.02%), Dinophyta (7.73%), Fungi (5.52%), Metazoa (jellyfish and copepods; 12.15%), undefined Stramenopiles and Eukaryota (2.76%).

Diversity recovery from a natural plankton assemblage can provide some indication whether the protocol was effective. From the Wuyuan Bay water sample, 181 good-quality sequences were obtained out of the 200 clones and deposited at NCBI under the accession numbers of KP099724–KP099904. From the result, 102 OTUs were found and designated as OTU1–OTU102 (S1 Fig). Most of the OTUs were composed of singletons, indicating no bias toward any specific taxon. With representative sequences from each OTU and their blast top-hits as references, a phylogenetic tree was constructed to aid in taxonomic assignment. Overall, a high diversity was found, including various lineages of phytoplankton and other plankton organisms such as ciliates, amoebae and fungi (Fig 5, Table 8). Among phytoplankton species, diatoms and chlorophytes were most abundant, while diatoms were highly diverse (Fig 5). Interestingly, we retrieved sequences of copepods and jellyfish although visually we did not see their presence in the water sample collected; most likely these were contributed by their eggs or larvae. Eight of the OTUs (group ④) appeared to represent *Ostreococcus lucimarinus*, as distances among themselves and with *O*. *lucimarinus* were very small (≤ 2%). This indicated the existence of *O*. *lucimarinus* in Wuyuan Bay, constituting the first report of its presence in the Chinese coastal waters to the best of our knowledge.

**Table 8:** The taxa composition revealed by Lowest Common Ancestors (LCA) and corresponding OTU numbers.

Group	Taxa (No. of OTUs)
Archaea	*Nitrosopumilus* (1)
Amoebozoa	Vannellidae (8)
Chlorophyta	Bathycoccaceae (2), Mamiellales (7), *Micromonas* (1), *Ostreococcus* (1), *Tetraselmis* (1), Ulvophyceae (1)
Ciliophora	Oligohymenophorea (1), Spirotrichea (1), Sporadotrichida (3), Tintinnida (2)
Bacillariophyta	*Chaetoceros* (6), *Chaetoceros pseudo-curvisetus*-like species (1), Coscinodiscophyceae (1), Cymatosiraceae (16), *Eucampia* (1), Mediophyceae (2), *Minidiscus conicus*-like species (3), *Nitzschia* (1), *Pseudo-nitzschia micropora*-like species (1), *Skeletonema* (1), *S*.*grevillei*-like species (3), *S*. *menzellii*-like species (5), *S*. *tropicum*-like species (1), *Thalassiosira* (2), Thalassiosirales (6)
Dinophyta	*Amphidinium carterae*-like species (1), *Dinophysis* (1), *Gyrodinium* (1)
Fungi	Agaricomycetes (1), *Cladosporium sphaerospermum* (1), *Exophiala jeanselmii* (1), marine fungus (1), *Rhodotorula* (1)
Metazoa	*Oithona* (3), Paracalanidae (2), *Pleurobrachia* (1)
Undefined	Stramenopiles (1), Eukaryota (8)

Some undocumented ribotypes were found. For instance, in the clade of dinoflagellates, OTU59, 61, 63 and 64 showed distance > 4% to their most closely related genus. OTU84 was assigned as marine fungus, but it was phylogenetically very distant from any fungi whose ITS sequences are available in GenBank. Furthermore, the undefined OTU93, 94, 95, and 96 were grouped together separately from any eukaryotic group and somewhat close to the Archaea. Whether they are a novel group of basal eukaryotes requires further investigation. To our surprise, we recovered two sequences that were assigned *Nitrosopumilus* (*E* value = 0, identity = 98%), an Archaeon living by oxidizing ammonia to nitrite and always found in non-coastal seawaters. Retrieval of archaea rDNA by using eukaryote-specific primers has also occurred in our previous work, indicating sequence similarity between eukaryotes and some archaea in this gene.

## Discussion

Accurate assessment of biodiversity of a phytoplankton assemblage and quantifying abundance of constituent species using PCR both relies on efficient extraction of DNA with good quality. To make the results comparable across different samples or between different laboratories, it is imperative to establish a protocol that has been verified to be efficient for different species. Currently many methods exist for DNA isolation and purification. Classical protocols were developed for model organisms (plant, mammals and bacteria), and their application to non-model organisms in most cases requires modifications. Phytoplankton comprise a wide range of microorganisms, from those without cell wall (e.g. naked dinoflagellates) to those with fortified cell walls, such as diatoms with silica frustules and thecate dinoflagellates with cellulosic theca. It is difficult to disrupt cells with regular chemical processing for some phytoplankton with fortified cell wall or very resilient cell membrane. Many methods have been used to disrupt cells before DNA isolation, including freezing-thawing [2], liquid nitrogen grinding [3], sonication [11] and bead-beating [5, 6, 7, 8, 9, 10]. However, the cell disruption efficiencies are often low for freezing-thawing and grinding in liquid N_2_, and sonication always results in severe DNA fragmentation. Among these, the bead-beating method was convenient and time-saving and it is increasingly employed in recent molecular researches concerning natural phytoplankton assemblages including metagenomics and metatranscriptomics [19, 20, 21, 22]. However, how to make it applicable to all phytoplankton species has not been reported.

It has been reported that various kinds of phytoplanktonic cells in water, soil and sediment samples could be disrupted with beads of different materials and sizes. However, the cells of some species can be easily disrupted in the lysis buffer (even before any mechanical disruption) and release DNA. If excessive amount of mechanical treatment (e.g. bead-beating) is applied, the DNA is very likely to be fragmented by the strong shear force produced by high-speed movement of the beads. On the other hand, some species do not have tough cell covering and 3-day incubation without bead-beating has proven sufficient to extract DNA completely (e.g. *Karlodinium veneficum* [12], *Pfesteria shumwayae* [13], and *Pfiesteria piscicida* [23]). In these cases, applying bead-beating to samples of this species can possibly damage DNA. In this study, we tried to solve the conundrum by first incubating the samples in lysis buffer long enough (3 days) to disrupt the “fragile” cells to release their DNA, and then applying bead-beating to the pellet after centrifugation, followed by mixing the two parts for further DNA extraction. Our protocol not only protected the DNA already released from damage by the subsequent bead-beating, but also allowed complete disruption of any intact cells remaining after the incubation. Our results demonstrated that the DNA amount increased markedly while its integrity was retained compared to a protocol without the bead-beating step. In fact, it was revealed that some seemingly “fragile” species (e.g. *Chlorella* spp.) were not totally broken without bead-beating, as DNA yield increased substantially after bead-beating.

As a way to assess DNA recovery efficiency of our protocol, we estimated cellular DNA content based on our DNA yield and the number of cells collected from the culture for DNA extraction, and compared it to flow cytometric results. For the 3 dinoflagellates, extraction-based estimates were lower than flow cytometric estimates, indicating 75.76–81.23% recovery rate. This reflects 18.77–24.24% loss, which is consistent to typical loss during harvesting and DNA purification. In our previous studies, we observed up to 20% cell loss and average recovery rate of DNA from DNA binding columns at about 80% (unpublished data). The combined effect can amount to about 36% loss. With these factors taken into consideration, our DNA extraction and recovery efficiency is within the expected range. For the other 4 species, it is unclear why extraction-based estimates of cellular DNA content were higher than that from flow cytometric analyses, but it was not due to inflated spectrophotometric DNA estimate that could happen as a result of low DNA purity, because the A260/A280 and A260/A230 ratios for our samples all indicated good DNA purity and qPCR using these DNA extracts as templates gave reasonably high efficiencies. The only other possible reason is that the strain of *T*. *pseudonana* used in our study as a standard might have a larger genome than 0.035 Gbp we set based on the genome report for another strain [16]. This suggests a need to establish proper standards for flow cytometric measurement of the wide range of algal genome sizes. Nevertheless, our result indicated that our protocol gave high DNA recovery rate for these species. As DNA extraction efficiency is crucial for quantification of target DNA or species abundance based on qPCR, future studies should endeavor to assess DNA extraction and recovery efficiencies.

Besides thorough cell lysis and efficient DNA extraction, quantitative studies of phytoplankton using molecular techniques also require high quality of the DNA preparations. The ratios of A260/A280 and A260/A230 reflect the purity of DNA by indicating contamination of protein, polysaccharides and other compounds. Ratios of 1.8–2.0 and 2.0–2.5, respectively indicate good quality. Impurities in DNA solution inhibit downstream experiments particularly in PCR [24, 25]. In our study, column filtration rather than ethanol/isopropanol precipitation was used for the purification of DNA as it helped remove PCR inhibitors such as polysaccharides.

Reasonable DNA integrity is another important requirement of DNA samples for quantitative applications. DNA integrity is hard to prove, but some evidence can be obtained. In the present study, gel electrophoresis showed high molecular-weight (> 10kb) DNA bands without smears typical of DNA degradation or extensive shearing, indicating good DNA integrity with adequate length for PCR amplification of most genes. In addition, qPCR using ITS specific primers further provided consistent quantitative results, suggesting good DNA integrity. Furthermore, the reasonably accurate recovery of the cell numbers of *A*. *fundyense*, *P*. *donghaiense* and *Chlorella* sp. mixed in the field sample indicated that the DNA extraction method developed in this study is suitable for quantitative analyses of field phytoplankton samples.

The high diversity of plankton retrieved from Wuyuan Bay field samples also lends support to the potential utility of our protocol for biodiversity and taxon-specific abundance studies on natural plankton assemblages. The detection of thick-walled dinoflagellates along with thin-walled phytoplankton and even the “naked” ciliates and amoebae demonstrated that our method was effective in disrupting cells otherwise difficult to break, while protecting DNA integrity.

rDNA usually possesses large numbers of copies in the genome and varying levels of sequence conservation in different regions of the gene, making it an ideal marker for studying diversity and relative abundance of microorganisms in the environment [26, 27]. We chose to use the ITS region because with high variability (hence taxon specificity) it has increasingly been demonstrated to be a good marker for target species detection [28, 29], fine-resolution phylogenetic analysis [30, 31] and DNA barcoding of phytoplankton [14, 32, 33, 34]. So far, molecular investigation of diversity of environmental microorganisms mostly relies on the SSU rDNA due to the existence of its large datasets in public databases such as NCBI and SILVA. In contrast, the amounts of ITS sequences increased slowly and account for only a small portion of all rDNA sequences [35]. Therefore, it is necessary and urgent to establish a large dataset of ITS sequences, especially for marine phytoplankton. The successful amplification of the ~1.5 kb ITS and adjacent regions of the 9 cultured species and the wide range of organisms in the field plankton sample suggest broad utility of the primer set (18ScomF-3end and com28SR2) as well as the protocol in future endeavors to grow the ITS database. The sequences obtained from the cultured species (9 species new in the ITS database in GenBank) and field sample add to the database some unique lineages. In this study, the OTU picking was based on the sequence similarity of 98% [17] which is commonly accepted for 18S rDNA. However, the ITS regions hold higher mutation rates, and therefore, whether lower similarity of ITS for OTU picking should be used still remains to be studied.

The investigation of diversity and abundance of prokaryotes by rDNA has already been broadly implemented from different environments [36, 37]. For prokaryotes cell abundance can be estimated and compared from measured rDNA copies in the environmental samples because most prokaryotes hold less than 10 rDNA copies per cell [38]. In contrast, this is impractical in eukaryotic phytoplankton due to the considerable variations in rDNA copy numbers between different species [39]. In our study, they could range from approx. 10–10^4^. Therefore, it is very likely to lead to severe over- or under-estimation in phytoplankton studies. One possible way to solve this problem is to evaluate accurately the rDNA copy number in most common phytoplankton species. Although it will be a huge amount of work, with the universal primers and an established protocol like the one reported here, it is feasible.

The biodiversity research on natural phytoplankton communities is currently dominated by pyrosequencing (e.g. 454 GS) [26, 27, 40, 41]. This high-throughput parallel sequencing technology requires high-quality DNA extraction from field samples, including complete extraction, high purity and good integrity [42, 43]. Our bead-beating method, demonstrated to be applicable in the preparation of DNA from field samples, will be useful for DNA preparation for pyrosequencing. Our DNA extraction method may prove useful for processing phytoplankton samples from wider ranges of habitats (e.g. dinoflagellate cysts and diatom resting spores in sediments).

## Conclusions

In this study, we developed an efficient bead-beating based method for DNA extraction of phytoplankton that could recover their diversities in natural assemblages quantitatively. Using this protocol, the DNA yield from the phytoplankton species included in this study was improved clearly, while its integrity was preserved because the DNA released in the lysis buffer during incubation was temporarily removed to avoid damage by bead-beating. Our results demonstrate that this method can be applied in field investigation of phytoplankton diversities.

## Supporting Information

S1 Fig The phylogram of sequences from field samples for OTU picking. The bootstrap values derived from the two methods were marked as NJ/ML and those larger than 50/0.50 were shown near the nodes. The sequences by bold lines were in the same OTU and the others (including those with asterisks) were OTUs comprised of singletons. “a” and “b” refer to samples.
(TIF)

S2 Fig Effects of incubation time on DNA yield. The same number of cells was used in each sample and the final volume of DNA extract was also the same. Shown are means of triplicate samples, with error bars indicating standard deviations (n = 3).
(TIF)
