# Supplementary figures and images for: An Improved DNA Extraction Method for Efficient and Quantitative Recovery of Phytoplankton Diversity in Natural Assemblages
Source: PLoS One. 2015 Jul 28;10(7):e0133060. doi: 10.1371/journal.pone.0133060 (PMC4517865; doi:10.1371/journal.pone.0133060)

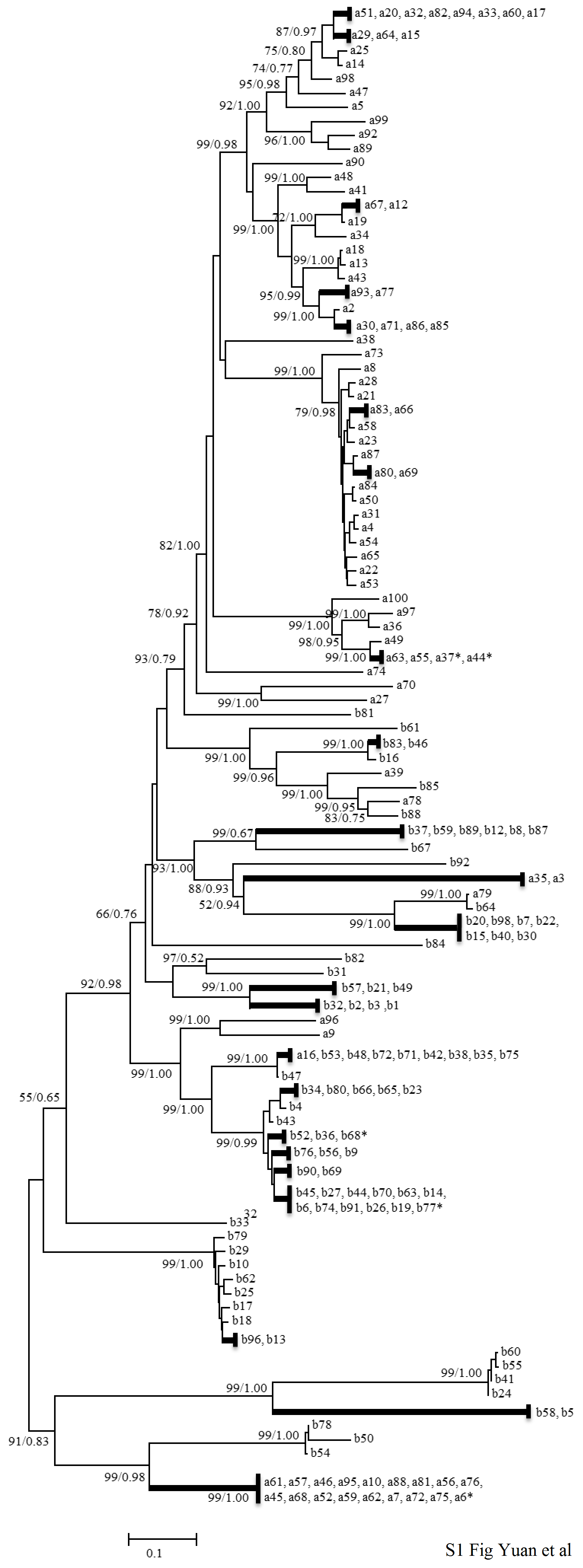

Supplement: S1 Fig — The bootstrap values derived from the two methods were marked as NJ/ML and those larger than 50/0.50 were shown near the nodes. The sequences by bold lines were in the same OTU and the others (including those with asterisks) were OTUs comprised of singletons. “a” and “b” refer to samples. (TIF) [file pone.0133060.s001.tif]
